# In vivo surface dose measurement using GafChromic film dosimetry in breast cancer radiotherapy: comparison of 7-field IMRT, tangential IMRT and tangential 3D-CRT

**DOI:** 10.1186/1748-717X-9-156

**Published:** 2014-07-15

**Authors:** Volker Rudat, Alaa Nour, Abdul Aziz Alaradi, Adel Mohamed, Saleh Altuwaijri

**Affiliations:** 1 Department of Radiation Oncology, Saad Specialist Hospital, P.O. Box 30353, Al Khobar 31952, Saudi Arabia; 2 SAAD Research & Development Center, Saad Specialist Hospital, P.O. Box 30353, Al Khobar 31952, Saudi Arabia

**Keywords:** Breast cancer, Intensity modulated radiotherapy, In vivo dosimetry, Surface dose

## Abstract

**Purpose:**

The purpose of this study was to compare the surface dose of 7-field IMRT (7 F-IMRT), tangential beam IMRT (TB-IMRT), and tangential beam 3D-CRT (3D-CRT) of breast cancer patients receiving adjuvant radiotherapy by means of in vivo GafChromic film dosimetry.

**Material and methods:**

Breast cancer patients receiving adjuvant radiotherapy of the whole breast or the chest wall were eligible for the study. Study patients were treated with a treatment plan using two different radiotherapy techniques (first patient series, 3D-CRT followed by TB-IMRT; second patient series, TB-IMRT followed by 7 F-IMRT). The surface dose was evaluated on three consecutive treatment fractions per radiotherapy technique using in vivo GafChromic film dosimetry. The paired t-test was used to assess the difference of in vivo GafChromic film readings or calculated plan parameters of the compared pairs of radiation techniques for statistical significance.

**Results:**

Forty-five unselected breast cancer patients were analysed in this study. 7 F-IMRT significantly reduced the surface dose compared to TB-IMRT. Differences were greatest in the central and lateral breast or chest wall region and amounted to a dose reduction of -11.8% to -18.8%. No significant difference of the surface dose was observed between TB-IMRT and 3D-CRT. A corresponding observation was obtained for the calculated skin dose derived from dose-volume histograms.

**Conclusions:**

In adjuvant breast cancer radiotherapy, 7 F-IMRT offers a significantly reduced surface dose compared to TB-IMRT or 3D-CRT.

## Introduction

Breast cancer is the most common cancer in females worldwide [1]. The majority of the patients will receive adjuvant radiotherapy after breast cancer surgery. The adjuvant radiotherapy after breast conserving surgery as well as the adjuvant radiotherapy after mastectomy of high risk patients have been shown to significantly improve local control and long-term survival [2,3].

Breast cancer radiotherapy is associated with acute and late radiation effects most importantly of the skin, heart and lung. Intensity modulated radiotherapy (IMRT) has the potential to improve dose homogeneity and conformity of a radiotherapy of the breast or chest wall thereby reducing side-effects [4-7]. Concerning skin toxicity, several studies have reported clinical benefit for IMRT of the breast [8-12]. The surface dose is of great relevance in breast cancer radiotherapy because it may have an impact on acute and late skin toxicity, cosmetic outcome and local control.

The purpose of this study was to compare the surface dose of 7-field IMRT (7 F-IMRT), tangential beam IMRT (TB-IMRT), and tangential beam 3D-conformal radiotherapy (3D-CRT) of breast cancer patients after breast conserving surgery or mastectomy by means of in vivo GafChromic film dosimetry. In vivo GafChromic film dosimetry was chosen because calculation of the superficial dose derived by treatment planning systems are known to be inaccurate in regions of electronic disequilibrium like the build-up region [13], and GafChromic films have been shown to be accurate and useful dosimetric tools for quality assurance in radiation therapy [14,15].

The superficial dose can be affected by multiple factors associated with the field arrangement like the source-surface-distance, field size, angle of incidence and wedges. In order to specifically evaluate the impact of the radiation technique on the superficial dose and to minimize the impact of other possible influencing factors, patients of this study were treated with a treatment plan using two different radiotherapy techniques, and in vivo GafChromic film dosimetric measurements were obtained from the two radiotherapy techniques used in the same patient.

## Methods

Patients with histologically confirmed breast cancer receiving adjuvant radiotherapy of the whole breast or the chest wall were eligible for the study. The primary goal of this study was to compare the surface dose produced by 7 F-IMRT, TB-IMRT, and 3D-CRT by means of in vivo GafChromic film dosimetry. The secondary goal was to compare calculated parameters of the dose distribution of the corresponding treatment plans.

For all patients, a treatment plan using two radiotherapy techniques was generated prior to radiotherapy. Radiotherapy technique 1 was applied for all radiotherapy fractions except for the last three radiotherapy fractions where radiotherapy technique 2 was applied. In vivo GafChromic film dosimetry measurements were performed during the last six radiotherapy fractions (three fractions technique 1 and three fractions technique 2). In the first series of patients, radiotherapy technique 1 and 2 consisted of 3D-CRT and TB-IMRT. In a second series of patients, TB-IMRT and 7 F-IMRT were used as radiotherapy technique 1 and 2. Table 1 demonstrates the prescribed fractionation regimen, treatment techniques and the timing of the in vivo GafChromic film dosimetry measurements.

**Table 1:** PTV, fractionation regimen, radiotherapy technique and timing of the in vivo Gafchromic film dosimetry

				**RT technique 1**		**RT technique 2**		**In vivo FD**
**PTV**	**n/p**	**D/f (Gy)**	**n/f**	**RT technique**	**Fraction number**	**RT technique**	**Fraction number**	**Fraction number**
Patient series 1: 3D-CRT versus TB-IMRT								
Whole breast	17 (7)	1.80	28	3DCRT	1-25	TB-IMRT	26-28	23-28
Whole breast	1 (1)	2.67	15	3DCRT	1-12	TB-IMRT	13-15	10-15
Chest wall	14 (7)	2.00	25	3DCRT	1-22	TB-IMRT	23-25	20-25
Patient series 2: TB-IMRT versus 7 F-IMRT								
Whole breast	7 (0)	1.80	28	TB-IMRT	1-25	7 F-IMRT	26-28	23-28
Whole breast	1 (0)	2.67	15	TB-IMRT	1-12	7 F-IMRT	13-15	10-15
Chest wall	5 (4)	2.00	25	TB-IMRT	1-22	7 F-IMRT	23-25	20-25

*Abbreviations*: *PTV* planning target volume, *n/p* Total number of patients (number of patients with left-sided breast cancer), *D/f (Gy)* Dose per fraction (Gy), *n/f* Number of fractions, *RT technique* Radiotherapy technique, *In vivo FD* In vivo Gafchromic film dosimetry, *3D-CRT* Conformal 3-dimensional radiotherapy, *TB-IMRT* Tangential beam intensity modulated radiotherapy, *7 F-IMRT* 7-field intensity modulated radiotherapy.

**Figure 1:**
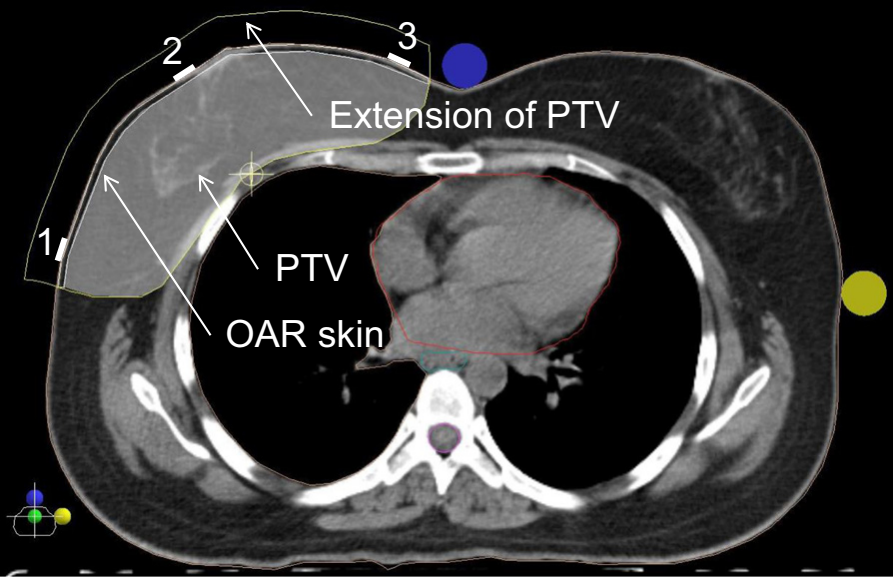
**Illustration of the OAR skin and the position of the in vivo Gafchromic dosimetry films using the image of a 7 F-IMRT plan in the isocenter plane.** The OAR skin was defined as a 3 mm strip from the surface of the skin to the PTV. PTV for the IMRT plans included the same PTV used for the 3D-CRT plans plus an extension into the air anteriorly of the skin of 1.5 cm to ensure appropriate opening of the 160 MLC Multileaf Collimator. The numbers denote the position of the in vivo Gafchromic dosimetry films (1 = lateral position, 2 = central position, 3 = medial position).

The target volumes were defined and the dose prescribed according to the International Commission on Radiation Units and Measurement (ICRU) Reports 50 and 62 recommendations. Accordingly, the target volume should be surrounded by the 95% isodose line of the prescribed dose. The planning target volume (PTV) definition for the whole breast or chest wall was done according to the recommendations of the breast cancer atlas for radiation therapy planning consensus definitions of the Radiation Therapy Oncology Group (RTOG) [16]. The PTV of the breast included the apparent computed tomography (CT) glandular breast tissue and the PTV of the chest wall the pectoralis muscle, chest wall muscles, and ribs. All PTVs excluded the outermost 3 mm from the superficial skin surface. The volume of outermost 3 mm from the superficial skin surface to the PTV was contoured as organ at risk (OAR) and labeled “skin” (Figure 1). The heart was defined as all visible myocardium, from the apex to the right auricle, atrium, and infundibulum of the ventricle. The pulmonary trunk, root of the ascending aorta, and superior vena cava were excluded. Other contoured structures included the ipsilateral and contralateral lung, the contralateral breast, the esophagus and the spinal cord.

The study was approved by the local Institutional Review Board and Ethics committee. All patients provided signed informed consent and the study was conducted in accordance with the ethical standards of the Helsinki Declaration of 1975, as revised in 2000. For the statistical analysis, the patient data were anonymized to guarantee privacy.

### In vivo GafChromic film dosimetry

**Figure 2:**
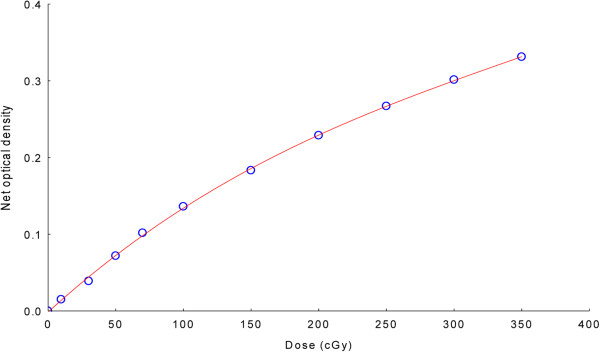
EBT3 film calibration curve.

In vivo dosimetry was performed using GafChromic EBT3 films (International Specialty Products, Wayne, New Jersey, USA). For calibration, ten 4 × 10 cm^2^ EBT3 filmstrips (lot # A05151202) were irradiated using the 6 MV photon beam of a Siemens Oncor Anvantgarde linear accelerator with a 160 MLC Multileaf Collimator at the center of 10 × 10 cm^2^ field. The films were irradiated at a depth of 10 cm and 90 cm source-surface distance (SSD) in a water-equivalent material phantom (PTW, Germany). Calibration film doses (0.0 Gy, 0.1 Gy, 0.3 Gy, 0.5 Gy, 0.7 Gy, 1 Gy, 1.5 Gy, 2.0 Gy, 2.5 Gy, 3.0 Gy, and 3.5 Gy) were calibrated against the ion chamber (Farmer chamber, 0.6 cc) measurement at the same location and depth. The output of the Oncor Anvantgarde linear accelerator was calibrated as per IAEA-TRS 398 protocol. Forty-eight hours after exposure, the ten calibration films plus an unexposed film from the same lot were scanned using a Vidar DosimetryPRO Advantage (Red) scanner (3D Systems Corporation, USA), read with Mephysto mc^2^ 1.6 (PTW, Germany) software, and optic density-dose calibration curves were obtained (Figure 2). Based on the calibration data, the film dose of the in vivo Gafchromic dosimetry films was estimated using the patient plan verification software VeriSoft (PTW, Germany).

For the in vivo dosimetry, 3 × 3 cm^2^ GafChromic films were carefully placed at three defined positions on the skin of the treated breast or chest wall. One film was placed at the central beam axis (“central”), the two other films 2 cm inside the medial and lateral treatment field borders (Figure 1). Films were scanned 48 hours after irradiation. For the dose estimation, ten point dose measurements were performed on a region of interest (ROI) of 2.5 × 2.5 cm^2^ and the mean dose calculated. Nine of the ten point dose measurements were performed at defined equally distributed positions and one in a random position. Dose readings were given as percent of the prescribed dose for all calculations.

The time interval of 48 hours between exposure and measurement of the calibration films and the in vivo dosimetry films was chosen because variations in net optical density have been shown to be negligible at this postirradiation development time [17], and because the time interval of 48 hours is convenient in the clinical setting considering the weekend.

### Treatment planning

A non-contrast CT-simulation was performed in the supine position on a carbon breast board with the ipsilateral arm up and head turned to the contralateral side. Radio-opaque wires were used to mark the clinical boundaries. A CT scan was performed using 5 mm slice thickness. The CT scanning reference point was defined using the CT simulation software Coherence Dosimetrist (Siemens Medical), and target volumes (PTV and OARs) using the software Coherence Oncologist (Siemens Medical). The 3D-CRT and IMRT plans were generated using the treatment planning system XIO 4.4 (CMS, Inc. of St. Louis, Mo, USA). A Siemens Oncor Anvantgarde linear accelerator with a 160 MLC Multileaf Collimator was used for the treatment. The leaf width was 0.5 cm at the isocenter. The dose calculation was determined using the “Superposition” algorithm. The beam energy of 6 MV was used for all 3D-CRT and IMRT plans.Dose volume histograms (DVH) of the PTV and OARs of the 3D-CRT and IMRT plans were generated and dose parameters compared. The OAR “skin” was defined as the volume of outermost 3 mm from the superficial skin surface to the PTV (Figure 1) and the “skin dose” as the calculated mean dose of the whole volume.

The Homogeneity index (HI) was defined as the fraction of the PTV with a dose between 95% and 105% of the prescribed dose. The Conformity Index (CI) was defined as the fraction of the PTV surrounded by the reference dose (V95%) multiplied by the fraction of the total body volume covered by the reference PTV dose ((PTV95%/PTV) × (PTV95%/V95%)) [18].

### 3D-CRT plans

**Figure 3:**
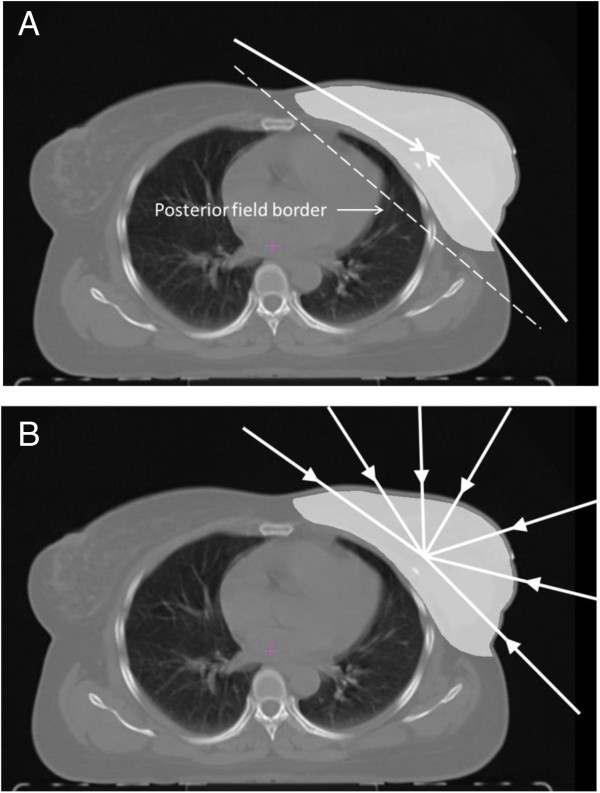
Illustration of the beam configuration used for 3D-CRT and TB-IMRT (A) and 7 F-IMRT (B).

The dose was prescribed to the ICRU reference point which was usually the isocenter located in the PTV volume centroid. Two tangential semi-opposed beams (to avoid divergence), physical wedges (usually 15° or 30°), a 160 MLC Multileaf Collimator, and 6 MV photons were used for 3D-CRT. The beam angles, wedge angles, and beam weighting (usually minimal) were chosen to optimize coverage of the PTV, while minimizing exposure to the ipsilateral lung, heart and contralateral breast. Gantry angles ranged from 42° to 55° for the medial fields and from 224° to 232° for the lateral fields for patients treated on the right side, and from 305° to 322° for the medial fields and from 133° to 147° for the lateral fields for patients treated on the left side (Figure 3).

### TB-IMRT and 7 F-IMRT plans

**Figure 4:**
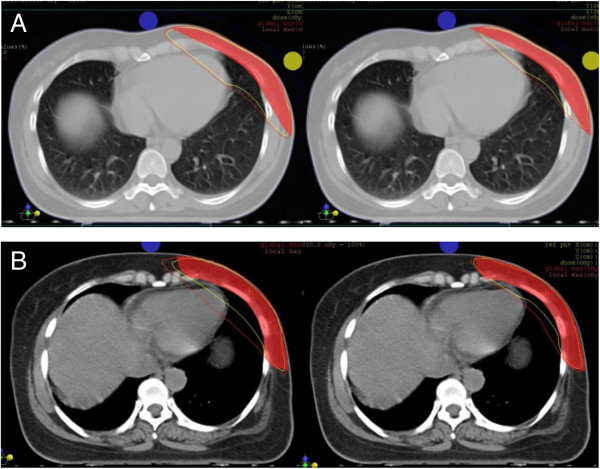
**Comparison of two radiotherapy plans of the same patient. (A)**, 3D-CRT versus TB-IMRT; **(B)**, TB-IMRT versus 7 F-IMRT. The yellow lines represent the 95% isodose lines, and the red line the 90% isodose lines.

The PTV for the IMRT plans included the same PTV used for the 3D-CRT plans plus an extension into the air anteriorly of the chest of 1.5 cm to ensure appropriate opening of the 160 MLC Multileaf Collimator (Figure 1). Inverse treatment planning and 6MV photons were used for all IMRT plans. The dose was prescribed to the PTV, and as initial dose volume constraints the IMRT prescription table provided by the XIO treatment planning system was used (Table 2). The IMRT plans were optimized to cover the PTV and spare the surrounding tissues as much as possible. A step-and-shoot technique was applied. An optimization with 100 iterations was then applied, and followed by a semiautomatic segmentation (minimum 3 cm step size). Segments equal or less than 2 MU were expelled from the plan. Tissue inhomogeneities were considered in the treatment planning optimization process, and the dose calculation algorithm used was “Superposition”. For the TB-IMRT plan, the same beam orientations and angles of the corresponding 3D-CRT plan were used. A hinge angle of approximately 190° between the first and last beams was used for the 7 F-IMRT plan with an angle separation of around 20° and avoiding normal incidence entry angles (Figure 3). For all techniques, plans were developed to deliver 95% of the prescribed dose to the full PTV, and to minimize dose to the OARs lung and heart. A typical dose distribution for 3D-CRT versus TB-TMRT and TB-IMRT versus 7 F-IMRT is demonstrated in Figure 4.

**Table 2:** Dose-volume constraints for IMRT plans

**Structure**	**Type**	**Rank**	**Objective**	**Dose (cGy)**	**Volume (%)**	**Weight**
PTV	Target	1	Maximum	5200	0	100
PTV	Target	1	Minimum	4900	100	100
Ipsilateral lung	Organ at risk	2	Maximum	2000	20	100
Ipsilateral lung	Organ at risk	2	Minimum	1200	30	100
Heart	Organ at risk	3	Maximum	4500	0	100
Unspecified tissue	Organ at risk	4	Maximum	4500	0	100

### Statistical analysis

Per patient, in vivo GafChromic film dosimetry readings were obtained at three defined positions (central, medial, lateral) of the PTV (whole breast or chest wall) during the use of two radiotherapy techniques (first patient series, 3D-CRT followed by TB-IMRT; second patient series, TB-IMRT followed by 7 F-IMRT). The mean dose in vivo GafChromic film dose measurements obtained at three consecutive treatment fractions was used for the statistical analysis (Table 1).

The paired t-test was used to assess the difference of in vivo GafChromic film readings or calculated plan parameters of the compared pairs of radiation techniques for statistical significance. Differences were considered statistically significant if the two-sided p-value was less than or equal 0.05.

The Pearson correlation coefficient was calculated to assess the degree of association between the in vivo GafChromic film dosimetry readings in the central, medial or lateral position of the PTV and the calculated skin dose derived from DVHs.

## Results

In vivo GafChromic film dosimetry measurements and calculated plan parameters comparing two radiotherapy techniques in the same patient were obtained of 45 unselected breast cancer patients (Table 1).

Table 3 demonstrates that 7 F-IMRT significantly reduced the in vivo GafChromic film dosimetry readings in the central and lateral film position of the PTV compared to TB-IMRT. No significant difference of the in vivo GafChromic film dosimetry readings was found between 3D-CRT and TB-IMRT. A corresponding observation was made for the calculated skin dose derived from DVHs.

**Table 3:** Surface dose by means of in vivo Gafchromic film dosimetry and calculated skin dose in dependence of the PTV and radiation technique

	**Surface dose**^**†**^						Calculated skin dose*	
	**Film position**							
**Radiation technique**	**Central**		**Medial**		**Lateral**			
**PTV**	**Mean**	**SD**	**Mean**	**SD**	**Mean**	**SD**	**Mean**	**SD**
Patient series 1: 3D-CRT versus TB-IMRT								
Whole breast								
3D-CRT	53.8	6.0	42.2	5.0	48.5	7.1	91.8	13.3
TB-IMRT	53.4	5.4	41.2	6.5	49.1	8.4	90.8	8.7
Difference (%)	-0.7		-2.4		1.2		-1.1	
p-value*	n.s.		n.s.		n.s.		n.s.	
Chest wall								
3D-CRT	58.1	5.7	47.2	9.0	49.8	5.1	92.2	6.0
TB-IMRT	58.3	6.3	45.4	7.9	51.9	6.2	92.0	8.8
Difference (%)	0.3		-3.8		4.2		-0.2	
p-value*	n.s.		n.s.		n.s.		n.s.	
Patient series 2: TB-IMRT versus 7 F-IMRT								
Whole breast								
TB-IMRT	54.0	3.9	45.0	5.6	41.7	4.5	86.8	6.9
7 F-IMRT	45.6	2.3	43.6	7.0	35.8	7.4	80.6	10.8
Difference (%)	-15.6		-3.1		-14.1		-7.1	
p-value*	<0.01		n.s.		0.05		0.03	
Chest wall								
TB-IMRT	60.1	5.3	39.4	12.0	59.2	3.5	95.2	2.3
7 F-IMRT	53.0	5.9	38.2	17.6	48.1	8.4	90.5	2.0
Difference (%)	-11.8		-3.0		-18.8		-4.9	
p-value*	0.02		n.s.		0.05		<0.01	

*Abbreviations:*^†^Proportion of the prescribed dose (%), *SD* Standard deviation, *Paired t-test, *n.s.* not significant, other abbreviations: see Table 1.

The correlation analysis revealed a week but statistically significant correlation between the calculated skin dose and the in vivo GafChromic film dosimetry readings in the central (r = 0.39; p = 0.01), medial (r = 0.28; p = 0.02) and lateral (r = 0.35; p < 0.01) film position of the PTV.

Comparison of plan parameters revealed a significantly improved CI and significantly reduced dose to the left heart and ipsilateral lung of TB-IMRT plans compared to 3D-CRT plans (Table 4). As expected, 7 F-IMRT plans exhibited a significantly increased heart and lung dose compared to TB-IMRT plans due to the multiple beam angles used. No significant difference concerning the CI and HI was observed between the 7 F-IMRT and corresponding TB-IMRT plans. It should be noted that patients treated with 7 F-IMRT were unselected concerning the thorax or cardiac geometry in this study.

**Table 4:** Relevant plan parameters of 3D-CRT versus TB-IMRT or TB-IMRT versus 7 F-IMRT of the breast or chest wall

**Organ**	**Patient series 1: 3D-CRT versus TB-IMRT**				**Patient series 2: TB-IMRT versus 7 F-IMRT**			
**Factor**	**RT technique**	**Mean**	**95% CI**	p-value*	**RT technique**	**Mean**	**95% CI**	p-value*
Skin								
Mean Dose (percent of the prescribed dose)								
	3D-CRT	92.0	(88.1; 95.9)	n.s.	TB-IMRT	90.0	(85.8; 94.2)	<0.01
	TB-IMRT	91.4	(88.1; 94.8)		7 F-IMRT	84.4	(78.6; 90.3)	
	Difference (%)	-0.7			Difference (%)	-6.2		
PTV								
Homogeneity index								
	3D-CRT	0.73	(0.65; 0.80)	n.s.	TB-IMRT	0.75	(0.68; 0.81)	n.s.
	TB-IMRT	0.73	(0.67; 0.78)		7 F-IMRT	0.72	(0.63; 0.81)	
	Difference (%)	0			Difference (%)	-4.0		
Conformity index								
	3D-CRT	0.76	(0.71; 0.81)	<0.001	TB-IMRT	0.88	(0.85; 0.91)	n.s.
	TB-IMRT	0.86	(0.82; 0.89)		7 F-IMRT	0.88	(0.85; 0.91)	
	Difference (%)	13.2			Difference (%)	0		
Heart (left-sided PTV)								
Mean Dose (cGy)								
	3D-CRT	506	(359; 654)	n.s.	TB-IMRT	824	(423; 1224)	0.01
	TB-IMRT	504	(380; 628)		7 F-IMRT	1647	(937; 2357)	
	Difference (%)	-0.4			Difference (%)	99.9		
V70%								
	3D-CRT	5.12	(2.49; 7.75)	0.03	TB-IMRT	9.25	(1.7; 16.8)	n.s.
	TB-IMRT	2.57	(1.06; 4.09)		7 F-IMRT	10.00	(1.5; 18.5)	
	Difference (%)	-49.8			Difference (%)	8.1		
Heart (right-sided PTV)								
Mean Dose (cGy)								
	3D-CRT	157	(33; 282)	n.s.	TB-IMRT	78	(60; 97)	<0.001
	TB-IMRT	149	(42; 282)		7 F-IMRT	704	(603; 805)	
	Difference (%)	-5.1			Difference (%)	802.6		
Ipsilateral lung								
Mean Dose (cGy)								
	3D-CRT	1276	(1179; 1374)	<0.001	TB-IMRT	1031	(842; 1221)	<0.001
	TB-IMRT	1036	(943; 1129)		7 F-IMRT	1535	(1382; 1689)	
	Difference (%)	-18.8			Difference (%)	48.9		
D60%								
	3D-CRT	194	(174; 214)	n.s.	TB-IMRT	139	(101; 177)	<0.001
	TB-IMRT	187	(168; 206)		7 F-IMRT	686	(458; 913)	
	Difference (%)	-3.6			Difference (%)	393.5		
D30%								
	3D-CRT	1327	(1036; 1618)	<0.001	TB-IMRT	934	(535; 1334)	<0.001
	TB-IMRT	922	(725; 1119)		7 F-IMRT	1775	(1459; 2091)	
	Difference (%)	-30.5			Difference (%)	90.0		
Contralateral lung								
Mean Dose (cGy)								
	3D-CRT	27	(24; 31)	n.s.	TB-IMRT	23	(18; 28)	<0.001
	TB-IMRT	26	(23; 29)		7 F-IMRT	328	(259; 397)	
	Difference (%)	-3.7			Difference (%)	1326.1		

*Abbreviations:95% CI* 95% confidence interval, *Vx%* percentage of tissue volume encompassed by the x% isodose line, *Dx%* dose to x% of the volume of the organ at risk, *Paired t-test, *n.s.* Not significant, other abbreviations: see Table 1.

## Discussion

The unique feature of our study is that patients were treated with a treatment plan using two radiation techniques (3D-CRT and TB-IMRT or TB-IMRT and 7 F-IMRT), and that the resulting superficial doses of the compared pairs of radiotherapy techniques were evaluated in the same patient. The advantage of this study design is that possible factors influencing the superficial dose (for example factors related to the patient set-up) are minimized and therefore observed differences are most probably related to the different radiation techniques.

Our in vivo GafChromic film dosimetry data show that 7 F-IMRT significantly reduces the surface dose compared to TB-IMRT or 3D-CRT in the adjuvant radiotherapy of breast cancer. A corresponding observation was obtained for the calculated skin dose derived from DVHs.

Our results are in excellent agreement with a film-based phantom study using an anthropomorphic female thorax phantom and GafChromic films for the dose measurements [19]. In agreement with our study, tangential irradiation resulted in surface doses mainly within 45–65% of the target dose (our study: 40-60%). 7 F-IMRT significantly reduced the surface dose compared to TB-IMRT or 3D-CRT. In the central beam axis the dose reduction was almost identical in both studies (approximately 15%; our study: 15.6%). Furthermore, no significant difference or only small differences were observed between 3D-CRT and TB-IMRT in both studies. It should be noted that the dose at the effective measurement depth of cells associated with acute skin reaction has been estimated to be approximately 15% below the surface dose measured by in vivo GafChromic film dosimetry [20].

There are also differences in the results of the phantom study and our study. In the phantom study, the greatest dose reduction was located in the regions closest to the medial and lateral field borders whereas in our study the greatest dose reduction was observed at the central and lateral beam region. This finding may be due to differences in the PTV and treatment set-up between the phantom and breast cancer patients.

However, the advantage of in vivo GafChromic film dosimetry compared to phantom studies is that in vivo measurements potentially more reliably reflect the clinical treatment situation with its uncertainties of patient positioning variability, breathing motion, and variability of the body geometry.

In agreement with the literature, comparison of calculated plan parameters showed an advantage of TB-IMRT compared to 3D-CRT concerning the CI, heart dose in patients with left-sided breast cancer and ipsilateral lung dose [6,7,21]. As expected, due to the multiple beam angles used, 7 F-IMRT showed a higher low-dose volume to the heart, ipsilateral and contralateral lung compared to TB-IMRT [22].

Thorough characterization of differences of the dose distribution is important to select the most appropriate radiation technique for the individual patient. For selected left-sided breast cancer patients with unfavorable thorax or cardiac geometry, multifield IMRT has been shown to be able to dramatically reduce the high-dose volume of the heart compared to 3D-CRT [4,5]. Multifield IMRT most probably reduces the risk of severe late heart toxicity and may therefore be the preferred radiation technique in these selected patients. In our study, unselected patients concerning the thorax or cardiac geometry were examined, and no significant sparing of the high-dose volume of the heart was observed. However, our data show that 7 F-IMRT is able to significantly reduce the superficial dose compared to TB-IMRT or 3D-CRT. Due to the multiple beam angles used, 7 F-IMRT is associated with a considerably larger low-dose volume to the heart and lungs compared to TB-IMRT or 3D-CRT. The advantage of a reduced risk of radiation skin reactions with 7 F-IMRT has to be balanced with the potentially increased risk of heart and lung toxicity. This applies especially to patients with left-sided breast cancer. Large breast size is significantly associated with an increased risk of radiation reactions of the skin [9,23]. For patients with large breasts 7 F-IMRT may be considered to reduce severe skin toxicity and to improve cosmetic results in particular in patients with right-sided breast cancer. In patients with inflammatory breast cancer where tumor cells are located in the skin, 7 F-IMRT may not be the preferred radiation technique due to the reduced superficial dose compared to TB-IMRT or 3D-CRT.

In conclusion, our data suggest that in radiotherapy of the breast or chest wall, 7 F-IMRT offers a significantly reduced surface dose compared to TB-IMRT or 3D-CRT.

## Competing interest

The authors declare that they have no competing interests.

## Authors’ contributions

AN, AA, AM and VR participated in the acquisition of the data, interpretation of the data and drafting of the manuscript. SM participated in the interpretation of the data and drafting of the manuscript. VR initialized and designed the study and performed the statistical analysis. All authors read and approved the final manuscript.
